# Gastrointestinal Lesions and Its Associated Factors in Adult Males With Iron Deficiency Anaemia: A Cross-Sectional Study From Tertiary Care Centre of North India

**DOI:** 10.7759/cureus.26905

**Published:** 2022-07-15

**Authors:** Aishwarya Singh, Rohit Mishra, Rajesh Ranjan

**Affiliations:** 1 Pathology, St. George's University, Grenada, GRD; 2 Community Medicine, Noida International Institute of Medical Sciences, Gautam Budh Nagar, IND

**Keywords:** endoscopy, males, inflammatory bowel diseases, gastrointestinal tract lesions, iron deficiency anaemia

## Abstract

Background

Around 30% of the world's population suffers from iron deficiency anaemia (IDA). The standard evaluation for IDA involves upper and lower endoscopy, which allows for the confirmation of pathology of the gastrointestinal tract (GIT) induced due to IDA through iron malabsorption mechanism or loss of blood. Assessing the prevalence of lesions of GIT of significant nature among males having IDA, was the goal of our study.

Methods

Our cross-sectional study was conducted for two years and involved 152 males (adults) with confirmed cases of IDA from the Outpatient (OPD) and In-patient (IPD) in the present hospital. Following collecting consent (both informed and written in nature), patient-specific data was collected in a standardized form, and a blood sample was taken for laboratory testing. The analyses were done at a 5% level of significance; an association was considered significant if the p-value < 0.05.

Results

The average age of the study participants was 59.6 years. The commonest lesions reported were antral gastritis (9.9%) and H. pylori gastritis (7.2%) in upper GI; and haemorrhoid (9.2%) and anal fissure (3.9%) in lower GI. The overall prevalence of any GI lesions was 65.1%. The GI lesions were significantly associated higher among men with age > 50 years (73.7%). The presence of occult blood in stools (p < 0.0001) and parasites in stools (p=0.0001) were significantly related to the presence of GI lesions.

Conclusion

GI lesions are frequently detected in males with IDA. Whether it is symptomatic male or asymptomatic male with anaemia refractory to iron treatment, GIT should be evaluated in them.

## Introduction

Around 30% of the world's population suffers from iron deficiency anaemia (IDA) [1]. IDA is extremely common in adults, despite being more prevalent in children and neonates. In India, IDA affects 53.2 % of non-pregnant women and 21.7 % of men [2]. According to age, gender, race, and ethnicity, the prevalence of IDA varies significantly [3]. Loss of blood due to menses and loss of iron during pregnancy results in the majority of IDA cases in premenopausal women. Low iron intake, insufficient iron absorption from the gastrointestinal tract (GIT), and chronic blood loss are all factors that contribute to iron deficiency [4]. However, occult bleeding from GIT causes IDA in males and women with menopause. A guaiac-based faecal occult blood test (FOBT) becomes positive with a daily loss of 10 mL of blood, while positive results for FOBT totally depend on the bleeding source site [5].

Among half of IDA patients, bleeding lesions in the GI tract are found. With a prevalence of 10%-17%, malignancies of GI are more frequent in older males and women with menopause with IDA [6,7]. In IDA patients, markers for GI malignancy are advanced age, male sex, increased serum lactate dehydrogenase (LDH), and reduced ferritin levels. Female gender, right-sided tumours, and a tumour diameter greater than 3 cm are all predictors of IDA in patients with colorectal cancer [3]. Although anaemia in IBD is mostly combined effect of IDA and anaemia of chronic disease (ACD), IDA alone remains a significant factor through reduced intake of iron owing to exclusion of food products which can aggravate symptoms of IBD; GI bleed (chronic); impaired iron absorption in Crohn's disease of the duodenum or upper jejunum; and an enhanced response of erythropoietin which is not paired with the accessible iron, leading to a non-effective erythropoietic process [8-10].

The standard evaluation for IDA involves upper and lower endoscopy, which allows for the confirmation of GIT pathologies that induced IDA through iron malabsorption mechanism or loss of blood. GIT (upper and lower) evaluation recommendations, on the other hand, are based mostly on data collected in a similar group of males and postmenopausal women [11-13]. Currently, there have been no published data in India on the burden of lesions of GIT in males (adults) having IDA, and the existing recommendations for GIT examination in this population are primarily based on the opinions of experts [11]. Assessing the prevalence of lesions of GIT of significant nature among males having IDA, was the goal of our study, as well as determining the factors (clinical and laboratory) for its occurrence.

## Materials and methods

Study setting and design

Our cross-sectional study was conducted for two years between June 2019 to May 2021, in the Pathology Department of a hospital in North India. Also, ethical board approval was obtained prior to the conduct of this study (Letter number NIMS/2019/04/223, Noida).

Study subjects and sample size

Our study enrolled laboratory-diagnosed cases of IDA male patients (Hb <13g/dL and 18 years or above) from the OPD and IPD as subjects. Patients were diagnosed as having IDA, if laboratory values showed at least serum iron concentration < 10 μg/mL with a transferrin saturation ≤ 20%, and/or mean corpuscular volume (MCV) < 80 fL and/or a serum ferritin concentration ≤ 30 ng/mL. A required size was determined to be 97, based on assumption, a 50% prevalence of lesions related to GIT among patients with IDA (as no specific related research was found by us in the Indian scenario) and a 10% absolute precision (absolute). Before patients were enrolled, consent (both informed and written) was got for every patient. Using a consecutive sampling procedure, a total of 152 patients participated in our study over the course of the study period. Patients with microcytic hypochromic anaemia other than IDA were excluded from the study.

Data and sample collection

The patients were subjected to clinical examination (signs) after obtaining a detailed history of diseases such as symptoms, prior medication and associated comorbidities and patient-specific data such as demographics and anthropometric measurements were recorded in a standard proforma. A blood sample (10 mL) was obtained for every patient for laboratory investigations such as reticulocyte count (RC), complete blood count (CBC), peripheral blood smears (PBS), total iron‑binding capacity (TIBC), serum iron (Fe), ferritin (Fer), liver and renal function (RFT and LFT), and lactate dehydrogenase (LDH). Stool samples were collected for examination of occult blood and ova, cysts. Also, computed tomography (CT) scans, x‑ray, ultrasonography (USG) for the abdomen, serum hepcidin levels, antibodies tests (anti‑tissue transglutaminase [anti-TTG] or anti‑gliadin, proctoscopy, endoscopy (both upper and lower GIT), and examination of bone marrow (including staining for iron), was done in selected patients as per indications.

Statistical analysis

All data were imported into an MS Excel document and analysed with SPSS version 21. Patients' baseline demographics, clinical profiles, and laboratory data were used to analyse the results. Continuous variables were reported as mean ± SD, whereas categorical data were represented as numbers and percentage (percent). The Kolmogorov-Smirnov test was used to determine whether the data were normal. Non-parametric tests were employed if normality was refused. In our study having a significant lesion of GI lesion was considered a dependent variable, and a comparison of independent variables (haematological findings, demographic, anthropometric details, clinical signs and symptoms, and medication and comorbidities) with the dependent variable was carried out using Chi-Square analysis. Statistical tests were conducted at a 5% significance level; any correlation was considered significant for a p-value < 0.05.

## Results

In our study, the average age of the study participants was 59.6 years, and 63.1% of subjects were above 50 years of age. The vegetarian dietary intake was observed in 64.5% of subjects. The mean BMI of the males with IDA was 21.9 kg/m^2^. A history of NSAID and PPI medication at least three months prior to the enrolment in the study was reported in 5.9% and 14.5% of subjects. Among 41.4% of men comorbidities such as hypertension (20.4%), diabetes (14.5%) and thyroid dysfunction (3.9%) were noticed (Table 1).

**Table 1:** Baseline characteristics of the study subjects. *Multiple responses BMI: Body mass index, NSAID: Non-steroidal anti-inflammatory drugs, PPI: Proton pump inhibitor

Variables	Number/Mean	%/SD
Age (in years)	59.6	20.9
Age group		
<50 years	55	36.1
>50 years	97	63.9
Diet		
Vegetarian	98	64.5
Mixed	54	35.5
BMI (kg/m^2^)	21.9	3.8
History of medication		
No	121	79.6
NSAID	9	5.9
PPI	22	14.5
Comorbidities		
No	89	58.6
Yes	63	41.4
If yes, comorbidity type*		
Hypertension	31	20.4
Diabetes	22	14.5
Thyroid dysfunction	6	3.9
Others	16	10.5

Among enrolled subjects, 69.1% reported to have any symptoms and 66.4% had GI symptoms. Upper GI symptoms were observed in 21.1% of subjects which included heart burn (13.8%) and epigastric pain (8.6%). The lower GI symptoms were observed in 45.4% of subjects, which included most frequently rectal bleeding (21.1%), constipation (20.4%), followed by change in bowel habits (5.9%) and diarrhoea (3.3%). The physical examination showed hepatosplenomegaly and epigastric sensitivity in 4.6% and 9.9% of subjects respectively (Table 2).

**Table 2:** Signs and symptoms among study subjects. *Includes GI symptoms, weight loss > 10 % in the previous year, abdominal pain, abdominal distention or bloating, weakness and easy fatigability ^$^ Multiple responses GI: Gastrointestinal

Variables	Number	%
Any symptoms*		
Yes	105	69.1
No	47	30.9
GI Symptoms^$^		
No	51	33.6
Yes	101	66.4
If yes		
Upper GI symptoms^$^	32	21.1
Heart burn	21	13.8
Epigastric pain	13	8.6
Lower GI symptoms^$^	69	45.4
Diarrhoea	5	3.3
Constipation	31	20.4
Change in bowel habits	9	5.9
Rectal bleeding	32	21.1
Physical examination findings		
Hepatosplenomegaly	7	4.6
Epigastric sensitivity	15	9.9

The haematological findings related to IDA showed the mean values for haemoglobin, MCV, ferritin, and transferrin as 9.86 ± 1.56 (g/dL), 75.8 ± 9.7 (fL), 6.9 ± 5.8 (ng/ ml), 7.2 ± 3.1 (g/L), respectively. The faecal examination showed occult blood in 36.8% of subjects and parasites/ova/cysts 13.8% of subjects (Table 3).

**Table 3:** Laboratory findings of the study subjects. MCV: Mean corpuscular volume

Laboratory parameters	Number/Mean	%/SD
Haematological		
Haemoglobin (g/dl)	9.86	1.56
MCV (fL)	75.8	9.7
Ferritin (ng/ ml)	6.9	5.8
Transferrin (g/L)	7.2	3.1
Faecal		
Occult Blood	56	36.8
Parasites/Ova/Cysts	21	13.8

The upper GI endoscopy was indicated in 55.3% of men with IDA. The findings showed that upper GI endoscopy was normal among 11.8% of subjects. Antral gastritis (9.9%) and H. pylori gastritis (7.2%) were the commonest lesions observed in upper GI endoscopy. The lower GI endoscopy was indicated in 41.4% of men with IDA. The findings showed that lower GI endoscopy was normal among 19.7% of subjects. Haemorrhoid (9.2%), anal fissure (3.9%) and colonic polyp (3.3%) were the commonest lesions observed in lower GI endoscopy. The endoscopy also showed GI lesions such as inflammatory bowel disease (1/152), colonic cancer (1/152) and gastric cancer (1/152) in men with IDA. Overall prevalence of any GI lesions was 65.1% among study subjects (Table 4).

**Table 4:** Upper and lower GI lesions among the study subjects. GI: Gastrointestinal

Lesion	Number	%
Upper GI endoscopy		
Not done	68	44.7
Done	84	55.3
If done, findings		
Normal	18	11.8
Antral gastritis	15	9.9
H. pylori gastritis	11	7.2
Duodenitis	8	5.3
Pangastritis	8	5.3
Coeliac disease	5	3.3
Gastric ulcer	4	2.6
Duodenal ulcer	3	2.0
Atrophic gastritis	3	2.0
Interstitial colitis	3	2.0
Gastric polyp	2	1.3
Erosive gastritis	2	1.3
Gastric cancer	1	0.7
Lower GI endoscopy		
Not done	89	58.6
Done	63	41.4
If done, findings		
Normal	30	19.7
Haemorrhoid	14	9.2
Anal fissure	6	3.9
Colonic polyp	5	3.3
Diverticulitis	4	2.6
Chronic colitis	2	1.3
Inflammatory bowel disease	1	0.7
Colonic cancer	1	0.7
Any GI lesion	99	65.1

The GI lesions were significantly associated higher among men with age > 50 years (73.7%) as compared to men having age < 50 years (26.3%). The BMI was higher among subjects (23.1 ± 5.2 kg/m^2^) with GI lesions as compared to those without GI lesions (20.3 ± 4.1 kg/m^2^). GI lesions were more common among vegetarians (58.6%) as compared to those having mixed dietary intake (41.4%). The subjects who had history of PPI medication, had higher chances of GI lesions (19.2%) as compared to those who had no PPI medication. Also, the patients with GI symptoms (75.8%) had higher chances of GI lesions as compared to those without any symptoms (24.2%). The presence of occult blood in stools (p<0.0001) and parasites in stools (p=0.0001) were significantly related to presence of GI lesions (Table 5).

**Table 5:** Association of the clinical, laboratory and baseline characteristics with the GI lesions among the study subjects. BMI: Body mass index, NSAID: Non-steroidal anti-inflammatory drugs, PPI: Proton pump inhibitor, GI: Gastrointestinal, MCV: Mean corpuscular volume

Variables	GI lesion [Number (%)]		P value
	Yes (n=99)	No (n=53)	
Age			
<50 years	26 (26.3)	29 (54.7)	0.0005
>50 years	73 (73.7)	24 (45.3)	
BMI (kg/m^2^)	23.1 ± 5.2	20.3 ± 4.1	0.0009
Diet			
Vegetarian	58 (58.6)	40 (75.5)	0.038
Mixed	41 (41.4)	13 (24.5)	
History of medication			
NSAID	6 (6.1)	3 (5.7)	0.920
PPI	19 (19.2)	3 (5.7)	0.028
Comorbidities			
No	57 (57.6)	32 (60.4)	0.738
Yes	42 (42.4)	21 (39.6)	
Any GI symptoms			
Yes	75 (75.8)	26 (49.1)	0.008
No	24 (24.2)	27 (50.9)	
Haematological			
Haemoglobin (g/dL)	8.6 ± 1.7	10.2 ± 1.4	<0.0001
MCV (fL)	74.9 ± 8.2	75.6 ± 7.8	0.610
Ferritin (ng/ml)	6.3 ± 5.7	5.9 ± 4.3	0.655
Transferrin (g/L)	8.3 ± 2.9	7.7 ± 3.5	0.260
Faecal			
Occult Blood	25 (25.3)	31 (58.5)	<0.0001
Parasites/Ova/Cysts	6 (6.1)	15 (28.3)	0.0001

## Discussion

A GI evaluation was performed on 152 males with IDA in this study. The majority of the subjects had significant GI lesions. The distribution was similar in the upper and lower GI tracts. The most prevalent GI lesions were antral gastritis and haemorrhoids. The presence of any GI symptom and the usage of a proton pump inhibitor were both linked to the presence of GI lesions.

In the current study, males with IDA had a 65.1 % (99/152) prevalence of GI lesions. Significant GI lesions were shown to be frequent in a homogeneous group of postmenopausal women and adult men, with a prevalence of 40%-70% in postmenopausal women and adult men and close to 50% in premenopausal women [14]. The commonest reason for IDA in postmenopausal females and males (adult) is GIT mucosal lesions that induce persistent blood loss, with GIT malignancy being common in this age group [15,16]. Loss of blood due to menses and malabsorption of iron owing to celiac disease and gastritis (atrophic or H. pylori) are the most common factors for IDA in premenopausal women [17].

Although the present study finding should be interpreted with caution due to the single-centric nature, the findings of our research recommend the investigation of GIT among adult males with laboratory-confirmed IDA. The commonest GIT lesion observed in the present study among patients was antral gastritis (9.9%). A similar study by Cook et al. showed commonest lesions of upper GIT included gastritis, esophagitis, ulcers (gastric or duodenal), and duodenitis, among both genders (male and female) of any age group having IDA [18].

H. pylori gastritis was found in 7.2% of the males with IDA in this study. H. pylori gastritis was an earlier identified factor for IDA, due to its capability for iron absorption impairment [19,20]. The link between H. pylori infection and IDA was established by demonstrating IDA reversal following H. pylori eradication [21]. Another prevalent finding in this analysis was celiac disease (3.3%), and the ratio of its proportion in the study to the proportion in the general population was 4:1, but an even greater frequency of 8.7% had previously been observed in IDA patients [22,23].

The most common lower GIT lesions detected in this investigation were haemorrhoids (9.2%), anal fissures (3.9%), and colonic polyps (3.3%). It has also been observed that haemorrhoids and anal fissures are prevalent lower GI lesions and that they are one of the main sources of rectal bleeding and may induce IDA [24,25]. Although just one subject (0.7%, 1/152) had inflammatory bowel illness, IDA is a common consequence of IBD. The prevalence of IBD revealed in the study by Goodhand et al. ranged between 16% and 68% [26].

Any GI-related symptom (p=0.008) was shown to be significantly associated with the presence of GI lesions in this study. A few studies have previously provided a correlation between any GI symptoms (heartburn, epigastric discomfort, diarrhoea, constipation, and changes in bowel habits) and the diagnosis of GI lesions [15,27]. In this study, the usage of PPI was associated with a higher percentage of GI lesion diagnoses (p=0.028). Since the reduced secretion of gastric acid is still clinically not proven as an attribute to IDA, this reflects such an association exists due to more frequencies of symptoms related to GIT, which necessitate the use of these medications, rather than the direct effect of gastric acid hyposecretion on iron absorption [28,29].

Limitations

This is the first local research that we are aware of that has identified GI lesions among men with IDA. The research is not without limitations. The sample size was limited and less heterogeneous because all of the participants were from a single institution. Furthermore, upper and lower GI endoscopy was not advised for all the patients in this study, and studies suggest that even individuals with stool occult blood negative can have GI lesions, thus researchers may overlook these GI lesions.

## Conclusions

GI lesions are frequently detected in males with IDA. Whether it is symptomatic male or asymptomatic male with anaemia refractory to iron treatment, GIT should be evaluated in them. The order of the assessment may be dictated by specific complaints. Despite the fact that nutrition-related deficiencies are a major causative reason for IDA around the world, just diagnosing IDA and not diagnosing the causative factors is not unacceptable. Significant attention shall be given to malignancy of GITs such as cancer of the colon or rectum, where IDA can be the only presenting symptom and diagnosis can be overlooked when the condition is not carefully investigated.
